# The Matrix in Filling Teeth

**Published:** 1887-05

**Authors:** 


ARTICLE VII.

### THE MATRIX IN FILLING TEETH.

The matrix is rapidly gaining favor, and we believe its intelligent use adds largely to the perfectness of many fillings, and renders operations that are difficult and tedious without its use, comparatively easy and more complete when finished. It gives an easy restoration of natural approximation, with unnecessary separations or wedging, and this is a great point gained. Many operators who experimented with the forms put upon the market at first, were not impressed with the benefits of the system. This result was occasioned partly by the imperfect form of matrices, but chiefly because they were not properly used, and the result was imperfect fillings at one or both of the lateral walls near the cervical border. The fault was in using instruments not adapted for reaching these points, and by the use of cohesive gold. Non-cohesive gold should be exclusively used, until the cavity is nearly full, and each piece of gold should be placed to extend from the lateral or cervical border to the interior of the cavity, and condensed firmly against cervical and lateral borders, and against the matrix and evenly carried up until the cavity is two-thirds full or even more, then finish with cohesive gold. In using the last pieces of non-cohesive do not condense thoroughly, and be sure that the first pieces of cohesive gold added are thoroughly malleted, and secured to the non-cohesive with a sharp pointed plugger.

Formerly the matrix was made only in forms to be secured between two teeth, but while these forms have been greatly perfected, there are now additional forms of matrices designed for a greater variety of cavities. Band matrices designated for teeth that are isolated, and for cavities in the buccal and lingual surfaces, are now made in a variety of forms, and all designated to facilitate the filling operation.

We desire especially to call attention to the admirable way in which the matrix supplements the efforts of the operator, when cement or amalgam fillings are used. In filling cavities with these plastics without the matrix, the filling placed in the deeper part of the cavity is displaced and pressed out by impaction nearer the orifice of the cavity, and the proper contour is obtained with difficulty, and at a sacrifice of solidity and proper joint at some point because there is no wall of resistance. With the matrix this is all obviated, and a more solid and properly contoured filling is the result. In using cement the surface of the matrix next the filling must be slightly rubbed with oil to prevent the cement adhering.

We consider the matrix especially adapted for these plastic fillings, and if the operator who does not use them will commence with their use in the plastics, he will not be able to get along without them; and while he will find that with gold greater care must be exercised, yet we are satisfied that he will find, that when they are rightly used he will accomplish in very many cases better results with less expenditure of time and brain, and with less pain and exhaustion to the patient.—*Western Dental Journal.*

---

### Editorial, Etc.

